# Insights into the Pathogenesis of Takotsubo Syndrome, Which with Persuasive Reasons Should Be Regarded as an Acute Cardiac Sympathetic Disease Entity

**DOI:** 10.5402/2012/593735

**Published:** 2012-10-15

**Authors:** Shams Y-Hassan

**Affiliations:** Department of Cardiology, Karolinska Institute, Karolinska University Hospital, Huddinge, 141 86 Stockholm, Sweden

## Abstract

The pathogenesis of takotsubo syndrome (TS) has not been established yet. The literature data dealing with the pathogenesis of TS are abundant but scattered among different medical specialities. Subarachnoid hemorrhage and other acute intracranial diseases and injuries are among the important and currently well-recognized trigger factors for TS. In both induced and spontaneous subarachnoid hemorrhages, signs suggestive of increased cardiac sympathetic overactivity have been documented. Surgical and pharmacological sympathectomy has shown to have protective cardiac effects in both animal and human studies. Increase in local release of norepinephrine from the heart of patients with TS has been measured. Signs of both cardiac sympathetic denervation and myocardial lesions adjacent to the cardiac nerve terminals have been seen. Furthermore, the systematized and typically circumferential pattern of ventricular wall motion abnormality is incongruent with the coronary artery supply region and appears most likely to follow the cardiac sympathetic nerve distribution. In conclusion, compelling literature data support the hypothesis that acute cardiac sympathetic disruption and norepinephrine seethe and spillover is causing TS in predisposed patients. TS is most probably an acute cardiac sympathetic disease entity causing myocardial stunning in which takotsubo is one among other cardiac image study findings.

## 1. Introduction

Takotsubo syndrome (TS), also known as neurogenic stunned myocardium, is characterized by a constellation of certain clinical findings: a clinical presentation mimicking that of acute coronary syndrome, a reversible typically regional ventricular wall motion abnormality with a peculiar circular pattern that does not match with the coronary artery supply region, and a coronary angiography showing no identifiable coronary artery culprit lesion to account for the observed regional ventricular wall motion abnormality [1–7]. The condition may occur in the setting of severe emotional stress, often after the sudden death of a loved one—hence the alternative name “broken heart syndrome” [3, 8]. Countless physical stress factors ranging from the most severe diseases to the most physiological processes may trigger the disease [5, 6, 9–11]. Among the diseases and injuries, which have provided a great contribution to the understanding of the pathogenicity of TS and are currently well-recognized trigger factors for TS, are the induced (animal), spontaneous (human) subarachnoid hemorrhage, brain death, and other intracranial processes [2, 12–22]. In this report, a mounting body of evidences supporting the hypothesis that the acute cardiac sympathetic firing and norepinephrine seethe and spillover being the cause of TS are presented.

## 2. Pathogenesis of TS

The etiology of TS has not yet been fully elucidated. Among the most frequently debated causal mechanisms underlying TS are the following: multivessel epicardial coronary spasm, myocardial microvascular dysfunction, aborted myocardial infarction caused by transient thrombotic occlusion of a long wrap-around left anterior descending artery, left ventricular outflow tract obstruction, blood-borne catecholamine cardiac toxicity, and cardiac sympathetic nerve firing with norepinephrine seethe and spillover [4, 23–27]. Literature data that, with compelling reasons, argue in favour of the causal link between the local cardiac sympathetic nerve terminal eruption and norepinephrine seethe and spillover and TS are vast.

## 3. Data Supporting the Involvement of Cardiac Sympathetic Disruption as a Cause of TS

The two main findings that solidly argue in favour of the involvement of the local cardiac sympathetic nervous system in causing the ventricular wall motion abnormality in TS are (A) a clear evidence of disruption of the local cardiac sympathetic nerve endings with local norepinephrine seethe and spillover into the ventricular myocardial tissue and (B) the characteristic systematized circular pattern of ventricular wall motion abnormality, which is unrelated to the coronary arterial system and appears most likely following the cardiac sympathetic supply system.

### 3.1. A: Cardiac Sympathetic Nerve Firing and Norepinephrine Spillover

There is unequivocal link between the state of mind and the development of TS. The frequent association of TS with sudden and intense emotional stress suggests that the mechanism of transient ventricular myocardial dysfunction might be sympathetically mediated [3, 5, 9–11, 28]. An emotional or a physical stressor has been reported in more than two thirds of patients with TS [9, 11]. There is sufficient data in both animal and human studies that indicate hyperactivation of the cardiac sympathetic nervous system in TS [2, 12–22, 29–31].

#### 3.1.1. Cardiac Sympathetic Hyperactivation in Animal Studies

Brain stimulation, induction of experimental intracranial hemorrhage, and stellate ganglion stimulation have caused ECG and myocardial changes reminding that of TS. These changes could be prevented either by surgical (spinal cord transection or severing) or pharmacological (reserpine, propranolol) sympathectomy. Porter et al. reported on persistent ECG abnormalities experimentally induced by stimulation of the brain in cats. The most common ECG findings reported in that study were inversion and increased amplitude of the T waves, deviation of the ST segment and prolongation of the QT interval. Such ECG changes are typically seen in patients with midapical ballooning type of TS. Interestingly, transection of the cervical spinal cord resulted in disappearance of the abnormalities and the return of the ECG to its prestimulation state [13]. Yanowitz et al. reported on unilateral and bilateral stellate ganglion stimulation and ganglionectomy in a series of experiments in the dogs. They showed that the electrocardiographic changes observed following unilateral alteration of sympathetic tone paralleled those electrocardiographic abnormalities seen in patients with lesions in the central nervous system [14]. Other investigators have shown that intracerebral injection of blood in mice caused myocardial lesions characterized by disruption and fragmentation of the affected myofibers in some and wide spread areas of myocardial degeneration and necrosis in others. They argued that the myocardial destruction may be mediated by wide spread release of noradrenaline at the local tissue level. These investigators wisely introduced the term “sympathetic storm” to indicate that the destruction was produced by the proximal release of noradrenaline from the adrenergic nerves of the myocardium [15, 32]. One other report studied the relationship of the nervous system to the “myocarditis” that occurs after adrenaline administration. The incidence of the myocardial lesions was significantly reduced in rats that had their spinal cords severed at C7-T1 prior to adrenaline administration. This indicated that it was not the administered adrenaline that caused myocardial lesions, but adrenaline was rather a trigger factor causing cardiac sympathetic disruption [16]. McNair et al. have reported that reserpine (a sympathetic blocking agent) pretreatment in mice subjected to simulated intracranial hemorrhage showed cardiac protection effect. This indicates that the sympathetic division of the autonomic system is involved in the mechanism responsible for myocardial damage following intracranial hemorrhage [33]. Novitzky et al. reported that surgical or pharmacological cardiac sympathectomy could prevent myocardial injury during brain death in the Chacma baboon [34]. Ueyama et al. have shown that left ventricular apical ballooning can be reproduced in rats subjected to immobilization stress, and this effect can be attenuated with adrenoceptor blockade [28].

#### 3.1.2. Cardiac Sympathetic Hyperactivation in Human Studies

A variety of intracranial diseases and injuries irrespective of the lesion localization may trigger the development of TS [2, 12, 20, 21, 35, 36]. This may occur through an increase in the intracranial pressure and hyperactivation of the cardiac sympathetic nervous system [22]. Beta adrenoceptor blockers have been shown to have cardioprotective effects in intracranial diseases and injuries in some studies [17]. Hammer et al. observed several electrocardiographic changes characteristic of intracranial diseases in a patient who underwent a successful operation of a basilar artery aneurysm. The electrocardiographic changes developed almost immediately with the manipulation of the circle of Willis and reverted in the same manner when the manipulation was discontinued. The rapid appearance and disappearance of the electrocardiographic changes with perturbations of the nervous system strongly suggests that these effects are due to neural rather than humoral factors [29]. Walter et al. in a study comparing treatment with adrenergic-blocking agents and placebo in patients presenting within 48 hours of subarachnoid hemorrhage showed that early adrenergic blockade (propranolol) benefits patients (particularly women) with subarachnoid hemorrhage for up to one year in terms of lesser neurological deficits [37]. Zhu et al. have reported on a patient with electroconvulsive therapy (ECT)-induced myocardial stunning with a clinical picture consistent with TS. The condition recurred during repeat ECT in spite of nitrate and calcium channel therapy. On the other hand labetalol (beta and alpha blocker) given intravenously to block the effect of sympathetic surge prevented the recurrence of TS during repeated ECT [38]. Bernstein et al. described a case with neurogenic stunned myocardium with signs consistent with TS in a patient with Guillain-Barre syndrome. This patient showed a severe reduction in heart rate variability and a shift toward a predominantly low-frequency autonomic modulation of heart rate, consistent with increased sympathetic tone, a vagolytic effect, or both [39]. Akashi et al. found decreased heart rate variability in 10 patients with TS compared with healthy controls. These results support the hypothesis that TS is caused by neurogenic stunned myocardium due to acute cardiac autonomic dysfunction [30]. Bonnemeier et al. tested the hypothesis that autonomic modulation might account for altered regional dysfunction in 37 patients with TS. The investigators used traditional and advanced heart rate variability to compare profiles in 27 patients with apical ballooning and 10 with midventricular ballooning pattern. They found that patients with midventricular ballooning had higher heart rate, greater low-frequency power, and greater low-frequency-to-high-frequency ratio compared to the patients with apical ballooning. These results indicate an increase in sympathetic tone affecting the sinus node in patients with midventricular ballooning compared to a lesser or no effect on the sinus node in the apical ballooning patients. The authors interpreted the results as representing differential activation of the left stellate ganglion and left cardiac sympathetic nerves in patients presenting with apical ballooning and the right stellate ganglion and right cardiac sympathetic nerves in patients presenting with midventricular ballooning [40]. TS has been reported after dobutamine administration for therapeutic or diagnostic purposes in many case and case series reports. Dobutamine is a sympathomimetic agent, predominantly a *β*_1_-adrenergic agonist, and has mild vasodilatory effects. The reports of Dobutamine-induced TS further implicate enhanced sympathetic stimulation in the pathogenesis of TS by demonstrating a causal link between beta-adrenergic stimulation and the development of TS [41].

#### 3.1.3. Cardiac Sympathetic Denervation

Iodine 123 meta-iodobenzylguanidine (^123^I-MIBG) is an imaging tracer that can be used to assess the cardiac sympathetic function. Endogenous norepinephrine and ^123^I-MIBG share similar myocardial uptake, storage, and release characteristics. The evidence of cardiac sympathetic denervation in regions with wall motion abnormalities in patients with TS detected by ^123^I-MIBG scintigraphy is substantial. Banki et al. studied 42 patients with subarachnoid hemorrhage with echocardiography, myocardial scintigraphy with technetium sestamibi (MIBI) and ^123^I-MIBG to assess myocardial perfusion and sympathetic innervation, respectively. All patients with interpretable scans (*N* = 41) had normal MIBI uptake. Twelve subjects had either global (*n* = 9) or regional (*n* = 3) absence of ^123^I-MIBG uptake. In comparison with patients with normal ^123^I-MIBG uptake, those with evidence of functional denervation were more likely to have regional wall motion abnormalities (92% versus 52%, *P* = 0.03) and cardiac troponin I levels >1 *μ*g/L (58% versus 21%, *P* = 0.029). The investigators concluded that left ventricular systolic dysfunction in humans with subarachnoid hemorrhage is associated with normal myocardial perfusion and abnormal sympathetic innervations. These findings may be explained by excessive release of norepinephrine from myocardial sympathetic nerve terminals, which could damage both myocytes and nerve terminals [18]. Burgdorf et al. studied 10 patients with TS with ^123^I-MIBG scintigraphy. They showed regional alterations in myocardial sympathetic innervation in the form of decreased heart to mediastinum ratio and increased washout rate. The study indicated impaired sympathetic neurotransmitter uptake in the hypokinetic/akinetic apical LV segments in patients with TS [42].

The cardiac sympathetic nerve endings have also been assessed by ^11^C hydroxyephedrine positron emissions tomography (PET) in a patient with TS. ^11^C hydroxyephedrine is a norepinephrine analog that provides a measure of cardiac presynaptic sympathetic activity. Prasad et al. studied a patient with TS with PET imaging with ^13^N ammonia (12.09 mCi) and ^11^C hydroxyephedrine (16.68 mCi). The tracer uptake was homogenous throughout most of the left ventricular myocardium on the ^13^N ammonia images, indicating preserved perfusion. Tracer uptake was, however, reduced in the majority of the midsegments and several of the apical and basal segments on the ^11^C hydroxyephedrine images, suggesting sympathetic abnormality [43].

#### 3.1.4. Cardiac Sympathetic Norepinephrine Spillover

Greenhoot and Reichenbach have reported on 9 patients with subarachnoid hemorrhage. All of them had cardiac lesions of varying degrees of severity that ranged from eosinophilia with preservation of cross-striations to disruption of myocardial cell cytoplasm with the formation of dense eosinophilic transverse bands (myofibrillar degeneration). Both the ECG changes and the cardiac lesions could be reproduced in cats given mesencephalic reticular formation stimulation. The investigators observed that the cell injury occasionally was seen adjacent to the nerves with more normal structure at a distance. Adrenalectomy did not protect the heart. They concluded that the ECG changes and the cardiac lesions were due to the release of catecholamines from adrenergic nerve endings in the heart [19]. Mertes et al. have demonstrated increased myocardial interstitial, but not plasma, norepinephrine release after brain death induction in pigs [44]. Kume et al. have also demonstrated elevated norepinephrine levels in the coronary sinus in 5 patients with TS, suggesting increased local myocardial catecholamine release [45]. Norepinephrine spillover from the cardiac sympathetic nerve terminals can decrease myocyte viability through cyclic adenosine monophosphate-mediated calcium overload, resulting histologically in contraction band necrosis which is one of the histopathological features of TS.

### 3.2. B: The Pattern of the Regional Ventricular Wall Motion Abnormality

In its typical form, the distribution of the left ventricular wall motion abnormality in TS has a strikingly regional and systematized pattern. It affects the ventricular myocardium circumferentially with a sharp transition between the affected (stunned) and the normal or hyperkinetic myocardium resulting in a conspicuous left ventricular ballooning during systole [46]. This sign has become the footprint of TS and was the reason for the introduction of the moniker takotsubo. Assessment of the systolic function of the left ventricle by two-dimensional speckle-tracking echocardiography has shown a circular systolic dysfunction in the acute phase of TS. Such pattern of systolic dysfunction is different from that caused by coronary vascular occlusion [47]. The heart is densely innervated with sympathetic nerves, which are distributed on a regional basis. The orderly distributed pattern of ventricular wall motion abnormality in TS appears to follow the sympathetic projections from the left and right stellate and caudal ganglia [48]. Zaroff et al. studied regional patterns of the left ventricular systolic dysfunction after subarachnoid hemorrhage in 30 patients. They observed that many of the wall motion patterns were atypical of coronary artery disease but correlated with the distribution of the myocardial sympathetic nerve terminals providing an indirect evidence for a neurally mediated mechanism of cardiac injury [20]. However, we should keep in mind that there are only two cardiac supply systems which are involved in the ventricular wall motion (kinesis) abnormality in a systematized and on a regional basis, and these are the coronary arterial and the neural supply systems. Impairment of cardiac conduction system may cause desynchronisation of the ventricular wall motion but does not lead to hypo-, a-, or dyskinesia. There are compelling evidences that challenge the fact that coronary arterial system is the cause of ventricular wall motion abnormality in TS. As a result, the only remained and likely cause for ventricular wall motion abnormality in TS is impairment in the neural supply system. Additionally, enormous data supporting the evidence of disruption of the cardiac neural (sympathetic) system have been presented in this paper. The different varieties of TS are most likely due to involvement of different branches of the cardiac sympathetic system in the disease process. Unfortunately, the anatomical distribution of the various branches of the cardiac sympathetic nervous system has not been mapped out yet in order to specify the branch or branches of the cardiac sympathetic nerves involved in TS. This may have facilitated categorization of the different varieties of TS (apical, midapical, midventricular, basal, focal, and global) according to the sympathetic branch involved in a manner similar to that of coronary artery occlusion causing anterior, inferior, lateral, and true posterior myocardial infarction.

## 4. Predisposing Factors

The emotional and physical stress factors are widespread during the day life among populations, but only a very limited number of patients develop TS. This indicates that there must be some predisposing factors that render some individuals more susceptible than others. TS has a special predilection for elderly postmenopausal women [9, 10]. Burgdorf et al. investigated the occurrence of malignancies in a cohort of 191 TS patients. They found that the prevalence of cancer was high (23.6%), which greatly exceeded the expected prevalence of cancer in age-matched populations in the United States (8.2%) and all European countries combined (7.8%) [49]. Sharkey et al. assessed prospectively the clinical profile and outcome of 136 consecutive patients with TS and showed that TS was a marker for increased noncardiac mortality [11]. The frequency of chronic anxiety disorders was significantly greater in patients with TS in another study [50]. Special chronobiological patterns of onset of TS have been observed by Citro et al. They showed seasonal (peak at summer and nadir at autumn) and diurnal (peak at the morning and nadir at the night) variation of the TS onset. This may indicate a potential link between seasonal and diurnal TS onset and the underlying pathophysiologic mechanisms [51].

## 5. Conclusion

Convincing data, supporting the hypothesis that the cardiac sympathetic nervous system is involved in the pathogenicity of TS, have been presented. The evidence for the local cardiac sympathetic nerve terminal eruption and norepinephrine seethe and spillover is enormous in TS patients. Moreover, the regional and systematized circular pattern of ventricular wall motion abnormality follows most likely the distribution of the different branches of the cardiac sympathetic nervous system. Consequently, persuasive reasons indicate that TS is an acute cardiac sympathetic disease entity causing myocardial stunning in which takotsubo is one among other cardiac image study findings.
